# Semi-Monolithic Detectors for TOF-DOI Brain PET: Optimization of Time, Energy, and Positioning Resolutions With Varying Surface Treatments

**DOI:** 10.1109/trpms.2025.3594103

**Published:** 2025-07-30

**Authors:** Fiammetta Pagano, Francis Loignon-Houle, David Sanchez, Nicolas A. Karakatsanis, Jorge Alamo, Sadek A. Nehmeh, Antonio J. Gonzalez

**Affiliations:** Instituto de Instrumentación para Imagen Molecular (I3M), Centro mixto CSIC-Universitat Politècnica de València, 46022 Valencia, Spain.; Instituto de Instrumentación para Imagen Molecular (I3M), Centro mixto CSIC-Universitat Politècnica de València, 46022 Valencia, Spain.; Instituto de Instrumentación para Imagen Molecular (I3M), Centro mixto CSIC-Universitat Politècnica de València, 46022 Valencia, Spain.; Department of Radiology, Weill Cornell Medical College, New York, NY 10065 USA.; Oncovision, 46022 Valencia, Spain.; Department of Radiology, Weill Cornell Medical College, New York, NY 10065 USA.; Instituto de Instrumentación para Imagen Molecular (I3M), Centro mixto CSIC-Universitat Politècnica de València, 46022 Valencia, Spain.

**Keywords:** Depth-of-interaction (DOI), Monte Carlo simulations, neural networks (NNs), semi-monolithic detectors, time-of-flight (TOF)-positron emission tomography (PET)

## Abstract

Semi-monolithic detectors, a hybrid configuration combining the benefits of pixelated arrays and monolithic blocks, present a compelling and cost-effective solution for positron emission tomography (PET) scanners with both time-of-flight (TOF) and depth-of-interaction (DOI) capabilities. In this work, we evaluate four LYSO-based semi-monolithic arrays with various surface treatments, read out with the PETsys TOFPET2 ASIC, to identify the optimal configuration for a novel brain PET scanner. The chosen array, featuring ESR on all surfaces except for the black-painted lateral pixelated ones, achieved 15.9 ± 0.6 % energy resolution and 253 ± 15 ps detector time resolution (DTR). neural network with multilayer perceptron architectures were used to estimate the annihilation photon impact position, yielding average accuracies of 3.7 ± 1.1 mm and 2.6 ± 0.7 mm (FWHM) along the DOI and monolithic directions, respectively. The comparative analysis of the four arrays also prompted an investigation into light sharing in semi-monolithic detectors, supported by a GATE-based simulation framework which was designed to complement the experimental results and confirm the observed trends in time resolution. By refining the detector design based on semi-monolithic geometry and optimized surface crystal treatment to enhance positioning accuracy, this study contributes to the development of a next-generation brain PET scanner, with competitive performance but at a moderate cost.

## I. Introduction

Positron emission tomography (PET) is the most accurate molecular imaging technique, but its spatial resolution still leaves room for improvement. The state-of-the-art conventional or total-body PET scanners feature a spatial resolution between 3.5–4 mm [1], [2], while organ-dedicated PET scanners achieve up to 1.5–1.8 mm [3], [4], [5], [6].

The most effective way to enhance image quality in PET is through precise determination of the line of response (LOR) and the interaction point along this. The former is achieved by enhancing the identification of the 3-D interaction position of the annihilation photons within the detector, while the latter relies on the detector’s precise timing capabilities, enabling the use of the time-of-flight (TOF) technique [7]. Consequently, there is an increasing interest in developing detector modules (and scanners) with both depth-of-interaction (DOI) and TOF capability [4], [8], [9], [10], [11], [12]. A tradeoff in 3-D positioning and TOF typically exists in those detectors, which is why efforts have been pursed to increase accuracy with two detector configurations: monolithic crystals and pixelated arrays. The advantages and limitations of both configurations have been extensively discussed in [9], [13], and [11]. In summary, pixelated arrays provide superior energy and time resolution, whereas monolithic crystals offer enhanced sensitivity and can achieve improved accuracy in determining the gamma-ray interaction position, including DOI, through analysis of the light distribution (LD). In pixelated arrays, planar spatial resolution is constrained by the crystal element size, and measuring the DOI typically requires additional hardware.

High spatial resolution is particularly required in the imaging of neurological structures, as PET has proven to be a pivotal tool in the diagnosis of neurological disorders, such as Alzheimer’s and Parkinson’s diseases [14]. One of the most widely used and commercially available brain PET scanners is the high resolution research tomograph (HRRT) [15]. The HRRT is based on pixelated LYSO crystals and features DOI but no TOF capability and has a limited sensitivity [16]. Over the past decade, newer prototypes also based on pixelated LYSO crystals, such as the VRAIN [6], Prism-PET [17], [18], NeuroeXplorer (NX) [4], and ultrahigh-resolution (UHR) [19], [20] have aimed to boost sensitivity, spatial, and time resolution. These high-performing systems, however, come at a high cost due to their fine crystal granularity (UHR), large fields of view (NX), or more complex geometry (VRAIN, Prism-PET). In contrast, the CareMiBrain [5] scanner offers a more affordable solution with a monolithic design, featuring a homogeneous spatial resolution due to the DOI information, though it lacks TOF capabilities.

In this work, we present the initial stages of the ultrahigh performance brain PET (UHB-PET) project, which aims to develop a brain PET scanner with enhanced sensitivity, impact positioning, and TOF capabilities while minimizing costs. To achieve this, the UHB scanner will utilize semi-monolithic arrays, a hybrid configuration between the pixelated and monolithic approaches [9], [21], [22], [23], [24], [25], [26], [27], [28]. This design consists of an array of 1×N slabs coupled to an array of M×M light SiPMs, where is shared along one direction (monolithic direction) and confined along the other (pixelated direction), combining the benefits of the two configurations. Semi-monolithic arrays are also suitable to be combined with a channel reduction readout [29], [30] which, by summing the signals in rows and columns, reduces the number of channels and so the power consumption. High sensitivity will be achieved by using 20 mm thick LYSO arrays with trapezoidal-shape to minimize the transaxial gaps, and by maintaining compact scanner dimensions (26 and 31 cm in axial and transaxial directions, respectively), a convenient solution to have wide solid-angle coverage. In order to reach the best tradeoff in terms of time, energy, and positioning resolution, four different surface treatments were evaluated for the LYSO crystals.

Although the main objective of this study is to compare different surface treatments, it also prompted an investigation into the intrinsic light-sharing properties of semi-monolithic arrays by integrating experimental results with Monte Carlo simulations.

## II. Material and Methods

### A. Materials

**Fig. 1.**
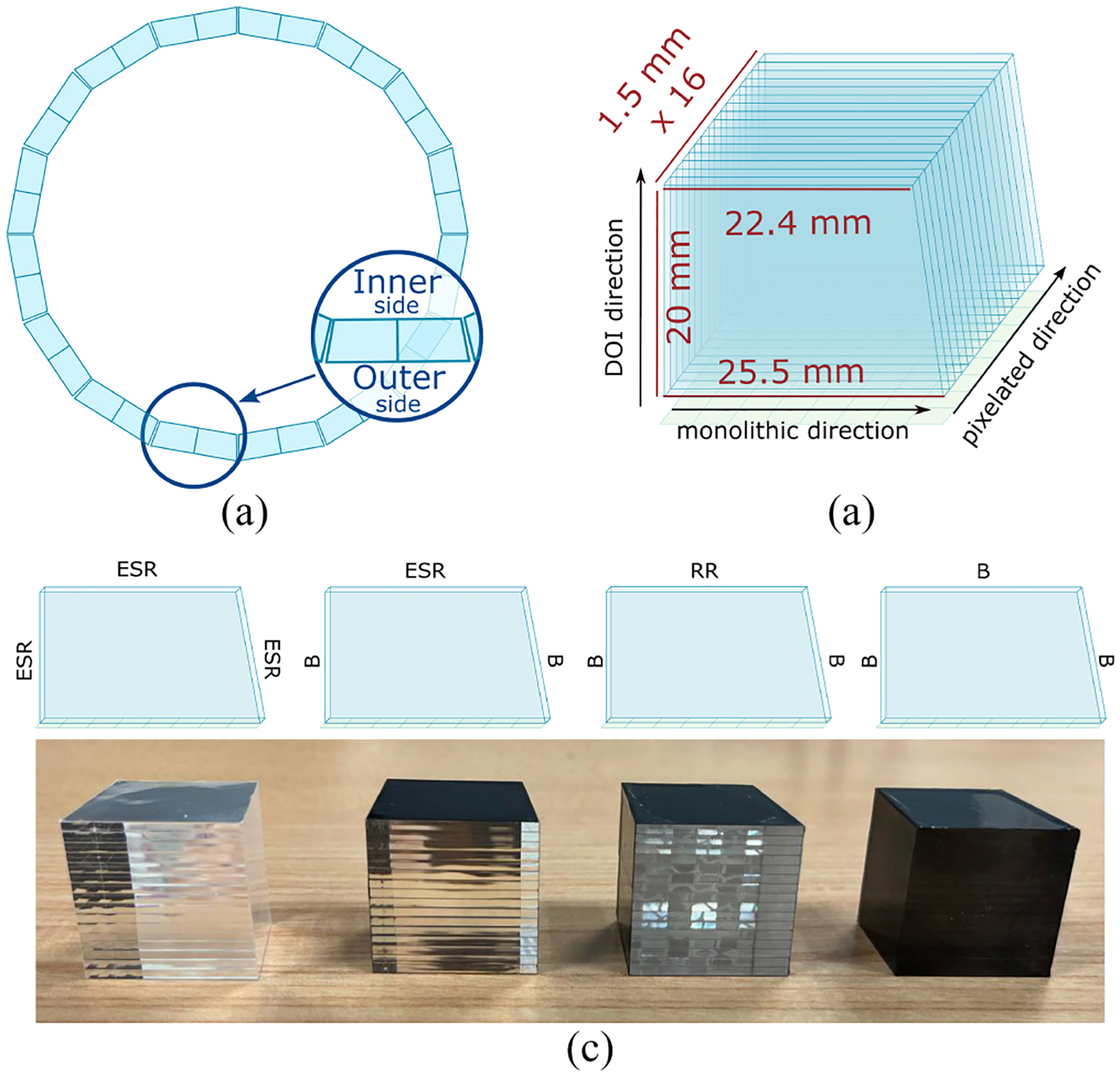
Detector design. (a) Geometry of a detector ring of the future UHB scanner. (b) Geometry and size of a single array. (c) Sketch and picture of the four arrays with different surface treatments. From left to right: All ESR (*All ESR*), ESR, and black painting (*ESR+B*), retro reflector and black painting (*RR*+*B*), and all black (*All Black*).

The detectors consist of arrays of 1×16 LYSO crystal slabs manufactured by EPIC Crystals (China). Each slab was polished after-cut and has trapezoidal shape, with an inner and outer side surface area of 22.4×1.5 mm^2^ and 25.5 × 1.5 mm^2^, respectively, and 20 mm distance between the inner-outer surface (see Fig. 1). The trapezoidal shape has been selected to minimize the transaxial gaps between adjacent detector blocks in the final system [26], [31].

In all cases, the slabs are separated by enhanced specular reflector (ESR) foils to maximize the optical isolation between them. The four arrays differ for the treatment of the external pixelated surfaces: both lateral and top pixelated faces covered with ESR (*all ESR*); the lateral pixelated faces black-painted and the top one covered with ESR (*ESR+B*); the lateral pixelated faces black-painted and the top one covered with retroreflector (*RR*+*B*); both the lateral and top pixelated faces black-painted (*All Black*). While ESR is a specular reflector, retroreflectors redirect light back to its source with minimal scattering, better preserving the original direction. All the arrays with pixelated lateral surfaces black painted were also wrapped with black tape for better stability and to preserve the black painting.

Each crystal array was coupled with optical grease (Bluesil paste 7) to an 8×8 array of SiPMs from Hamamatsu (S13361–3075AE-08), each with an active area of 3×3 mm^2^. Each SiPM was therefore collecting light from two adjacent slabs.

The TOFPET2 ASIC from PETsys electronics [32] was chosen for the electronic readout, since it is commercially available and scalable at system level [26], [30], [33], [34]. Each ASIC has 64 independent channels, each provided with signal amplification circuit, charge integration, analog to digital converters (ADCs), time to digital converters (TDCs), and three discriminators (*vth*_*t*1_, *vth*_*t*2_, and *vth*_*e*_). The first discriminator, *vth*_*t*1_, is used at low voltage to measure the first timestamp; the second, *vth*_*t*2_, is used at higher voltage to discard dark counts and for a first event validation; the third, *vth*_*e*_, is used for energy validation. Only events meeting the requirements of all three discriminators are accepted and digitized [35], [36].

### B. Experimental Measurements

All measurements were carried out using a ^22^Na source (0.25 mm diameter and 185 kBq activity) and acquiring in coincidence with a reference detector (20 ns coincidence time window). This was a 2×2×10 mm^3^ LYSO:Ce:Ca crystal with a read-out chain analogous to that described for the semi-monolithic arrays, having a 142 ps detector time resolution (DTR). Both the array under test and the reference crystal were encapsulated in light-tight black boxes and kept at a constant temperature of 20.0 ± 0.5 °C using air cooling.

For optimized performance, we performed scans of the bias voltage (*V*_*b*_) and thresholds (*vth*_*t*1_, *vth*_*t*2_, *vth*_*e*_) to find the combination providing the best compromise in terms of slab resolvability, time, and energy resolution [35], [37], [38]. The scan was repeated for each array, as the different surface treatment was expected to affect the optimal parameters. First, we kept fixed *vth*_*t*2_ and *vth*_*e*_ while varying *V*_*b*_ and *vth*_*t*1_, as the latter are known to have stronger effects on the performances of the detector. After choosing the optimal combination of *V*_*b*_ and *vth*_*t*1_, we analyzed the scan over *vth*_*t*2_ and *vth*_*e*_.

Each array was first measured in frontal and uniform irradiation—the same configuration in which the final system will operate—to assess its energy and time resolution performance.

**Fig. 2.**
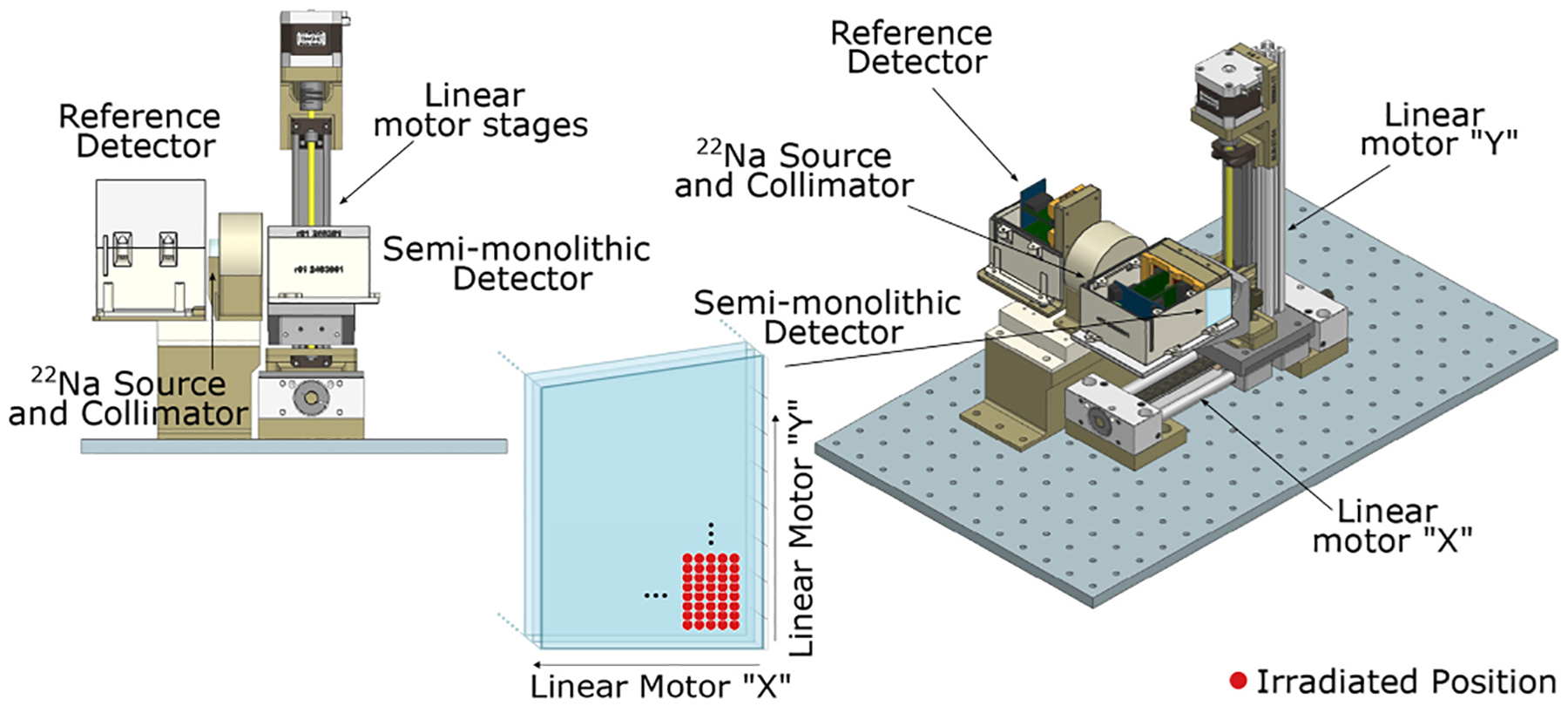
Sketch of the experimental setup for the side and collimated irradiation configuration.

Afterwards, measurements were conducted using collimated and side irradiation. For this configuration, a tungsten pinhole collimator with a diameter of 2 mm and a thickness of 3 cm was employed along with two linear motor stages (2-D) moving in steps of 1 mm (see Fig. 2). Data were acquired at each position for 20 min, ensuring a minimum of 500 events after photopeak selection and data filtering as described later in Section II-D. These measurements enabled the evaluation of positioning resolution both along the DOI and monolithic directions, while the resolution along the pixelated direction is limited by the slab thickness. Additionally, the time resolution as a function of the impact position was analyzed, providing valuable insights into light propagation in semi-monolithic arrays.

### C. Time and Energy Resolution

#### 1) Energy Resolution:

The energy resolution of each array was evaluated using all events, regardless of the slab where the interaction occurred. However, a variation of approximately 10% in the photopeak position was observed across slabs, requiring the application of gain equalization for optimized performance. Next, the photopeak of the energy spectrum was fitted with a single Gaussian function, and the energy resolution was defined as the full-width at half maximum (FWHM) divided by the centroid position. The error in the energy resolution was estimated as the standard deviation of the energy resolution measured for individual slabs.

Photopeak events were selected as those within ± 2*σ* of the centroid, and all subsequent analyses were performed on this subset of events.

A measurement in single mode was performed to have a preliminary estimation of the photosensor saturation. Because the saturation was found to be consistent across all arrays—attributable to a balance between the effects of different surface treatments and bias voltage—the energy resolution of the four arrays could be compared without applying a saturation correction.

#### 2) Time Resolution:

Although the TOFPET2 ASIC sorts by default each event by energy, it is also possible to sort the hits by time of arrival. To explore this, we compared the timing performance of the detectors using both sorting methods. When working with semi-monolithic arrays, because of the light sharing among multiple SiPMs and for reasons of ordered statistics, the best time estimator is not necessarily given by the first timestamp (neither the most energetic nor the fastest). A weighted average of the first few timestamps, using the energy collected by each corresponding hit as the weighting factor [35], [38], was performed to find the best timing estimator.

Before evaluating the time resolution, a few corrections were applied. First, the skew or time offset calibration: even if each channel is synchronized with the reference clock, they can show relative offset due to several factors, such as differences in internal path lengths. The calibration was done with a dedicated measurement using a pixelated array in a one-to-one coupling configuration with the photodetector, as described in [35] and [38].

As a consequence of the light sharing and different numbers of photons collected by each fired SiPM, the signal produced by each of them can show different amplitude and the measured timestamps will be affected by time walk. A time walk correction was applied by studying the correlation of the time difference between the reference and the *i*th-timestamp of the semi-monolithic array with the charge collected by the corresponding SiPM [35], [38].

By sorting the events by time of arrival, a correlation was found between the satellite peaks (a known draw-back of TOFPET2 ASIC by PETsys related to the internal clock when using a low value for time threshold [37]) and the time delay between the first and second fastest hits. Therefore, only events with such time difference below 1 ns were considered (about 70 % of all photopeak events) to filter out the majority of events falling under the satellite peaks.

After applying the above time corrections and filtering, we evaluated the energy-weighted average between the first *i*-timestamps, with *i* ranging from 1 to 8, the number of SiPMs coupled to one slab. For each of these estimators we computed the time difference with the reference and extracted the FWHM of the resulting distribution. The DTR of the semi-monolithic array was finally evaluated by quadratically subtracting that of the reference (142 ps).

Similar to the energy resolution, the time resolution was evaluated using all events, regardless of the slab where the interaction occurred. In this case as well, the error in the time resolution was estimated as the standard deviation of the time resolution measured for individual slabs.

### D. Impact Position Estimation

The impact position along the monolithic and DOI direction was estimated using supervised neural networks (NNs) techniques with a multilayer perceptron (MLP) architecture [39].

Before feeding the MLP with the input data, they were first preprocessed and filtered. The energies measured by the 64 channels for each event were summed in rows and columns (pixelated and monolithic directions) reducing them to 16 (8 + 8) signals to simulate the output of the channel reduction readout [30] which will be implemented in the final system. The pixelated, monolithic, and DOI coordinates were pre-estimated using analytical algorithms based on the scintillation LD data. The pixelated coordinate was estimated using the Center of Gravity (CoG) method [40], while the monolithic coordinate with the raised-to-second-power (RTP2) method, which reduces edge effects [41], [42]. For DOI estimation, the *E/I*_max_ approach was applied, calculating the ratio between total light collected and the maximum light intensity detected by a single SiPM [43].

To enhance the performance of the MLP, a more refined event selection was implemented compared to the one used for time resolution analysis, with the aim to maximize the rejection of multicompton events [39], [44].

1. Only events occurring in the third and fourth slab—those coupled to second SiPMs row—were considered. We chose the second SiPMs row instead of the first one to minimize edge effects (slab filter).
2. After applying the slab filter, events within ± 15% from the photopeak were selected (energy filter).
3. Finally, the events falling within the central 90 % of the RTP2 and *E/I*_max_ distributions were kept (position filter).

The data were then split into training, validation and test dataset according to the proportion 70%, 10%, 20%.

Two separate but analogous MLPs were used to estimate the position along the monolithic and DOI direction. The MLP architecture consisted of two hidden layers with 64 nodes each, followed by ReLU activation functions, and a final single output node for regression positioning (DOI or monolithic coordinate). The networks were trained using Adam optimizer and a mean square error loss function.

The inputs of the MLP in its standard configuration consist in the 16 energy signals. However, previous work showed that the inclusion of engineered features can improve the NNs performance [25], [45]. The MLP was therefore designed to handle a variable number of inputs to investigate the effect of adding different engineered features. Specifically, we used the pre-estimated impact position [25] using the CoG, RTP2, and *E/I*_max_ algorithms, the variance of the charge measured by each signal [45] and the average time difference between the fastest and the most energetic hit, since a position dependence was observed in our data.

The positioning accuracy was evaluated using the predicted positions of the test dataset, measuring the FWHM and the mean absolute error (MAE)

(1) $$\text{MAE}=\frac{1}{N}\times \sum_{i=1}^{N}\left|x_{\text{predicted}}\left(i\right)-x_{\text{real}}\left(i\right)\right|$$

with *x* being the coordinate along the monolithic or DOI direction. The FWHM provides the resolution of the predicted distribution, while the MAE provides the accuracy of the predicted value compared to the real one.

### E. Monte Carlo Simulations

To complement the experimental measurements, we used GATE v9.3 [46] to perform Monte Carlo simulations of the irradiation with 511 keV photons of a modeled 16-slab detector array as depicted in Fig. 1(b). The LUT Davis model was used for optical photon interactions at LYSO and glued-ESR surfaces, and the array was coupled with an optical medium to a sensitive surface representing an 8×8 SiPM array. Optical photon propagation was tracked and hits were recorded, followed by post-processing to incorporate single photon time resolution (SPTR) blurring, photon detection efficiency (PDE) photon removal and dark counts background. A single photon response was then applied for each hit, which were then summed to represent pile-up and generate one waveform per SiPM channel. The crossing time above one amplitude-equivalent photon response was recorded for each waveform—incorporating some electronic noise by fluctuating randomly the threshold with 10% jitter of the single photon amplitude—and the waveform was integrated to obtain the energy. An energy spectrum was created, events within 2*σ* of the photopeak were selected, and coincidences were created with random timestamps having a DTR of 142 ps to represent the reference detector. Note that because of the unknown exact values of some parameters, such as SPTR and equivalent TOFPET2 ASIC threshold amplitude, we had to used approximate values that enabled good comparison with experimental values. Nevertheless, the goal with those simulations was to assess timing resolution trends and differences rather than exact values.

## III. Results

### A. Time and Energy Resolution in Front Irradiation

The optimal combination of PETsys parameters (*vth*_*t*1_*, vth*_*t*2_*, vth*_*e*_) and bias voltage (*V*_*b*_), providing the best balance between slab resolvability, energy, and time resolution for each crystal array is summarized in Table I. Some general trends can be observed: when black-painted surfaces are used or when ESR is replaced by RR, *V*_*b*_ must be increased. This is because fewer photons reach the SiPM, resulting in a lower signal amplitude. To maintain optimal time performance, *vth*_*t*1_ must be reduced for these cases. Similarly, *vth*_*t*2_ and *vth*_*e*_ should also be lowered to account for the smaller signal amplitudes.

**Fig. 3.**
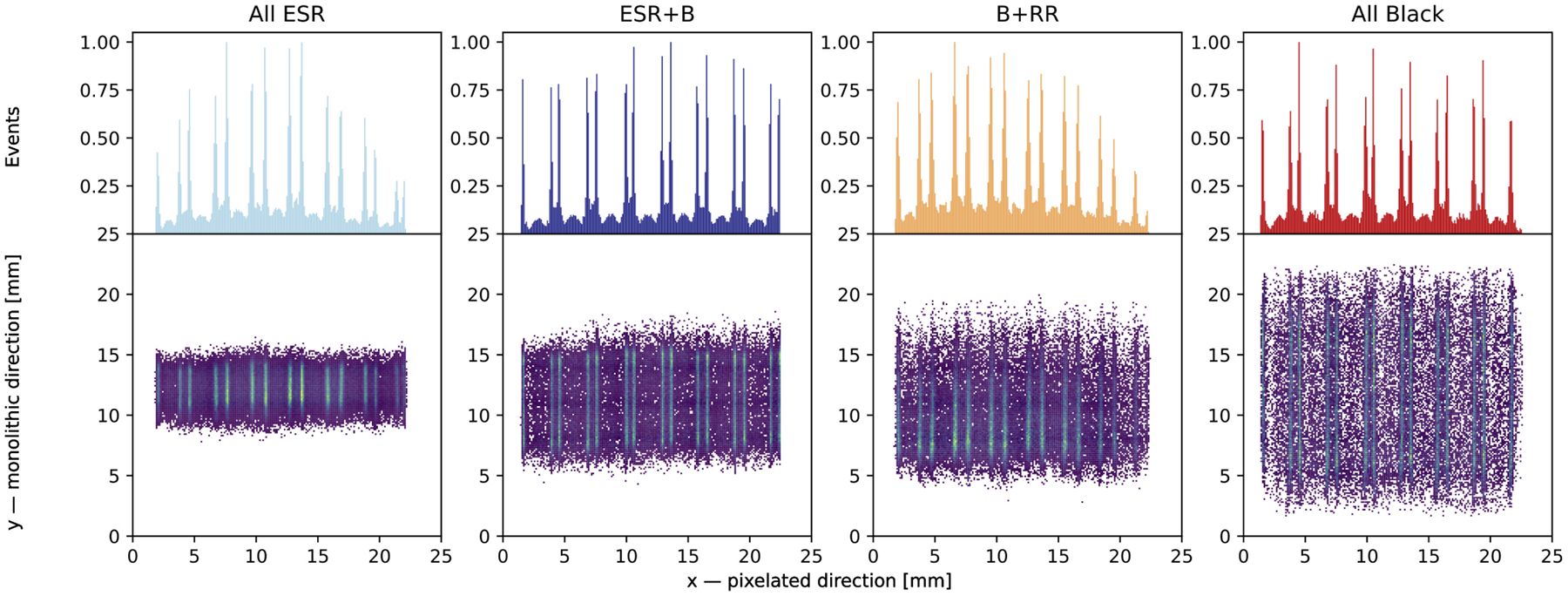
Bottom: 2-D histogram of the interaction position reconstructed along the pixelated and monolithic direction, using the the COG (pixelated) and RTP2 (monolithic) methods. Top: projection along the monolithic direction showing the slab resolvability.

Fig. 3 (bottom row) shows the reconstructed 2-D interaction position along the pixelated and monolithic directions obtained using the CoG and RTP2 method, respectively. Additionally, the top row shows the corresponding projections along the monolithic direction. In terms of slab resolvability, the four arrays show comparable performance, with an average background level ranging between 0.15 and 0.2, normalized to the maximum intensity. For the *all ESR* and *RR+B* arrays, some nonuniformity in slab intensity is observed. This is due to the source being inadvertently positioned too close to the array, resulting in nonuniform irradiation of the slabs. Nevertheless, the source position does not affect the energy or time resolution.

Observing the 2-D histograms in Fig. 3, from right to left one can notice an increasing compression along the monolithic direction. This behavior is attributed to the so-called edge effect: due to the finite size of the slabs, the light is reflected by the inner surfaces, producing a truncation in the LD which results in a shift of the interaction position toward the center of the crystal. The effect is stronger in the *all ESR* array, where all the crystal faces except the one coupled to the photosensor are covered by this specular reflector. By black-paining the lateral pixelated surfaces, the LD is better preserved, enabling better positioning capability (see Section III-C).

**Fig. 4.**
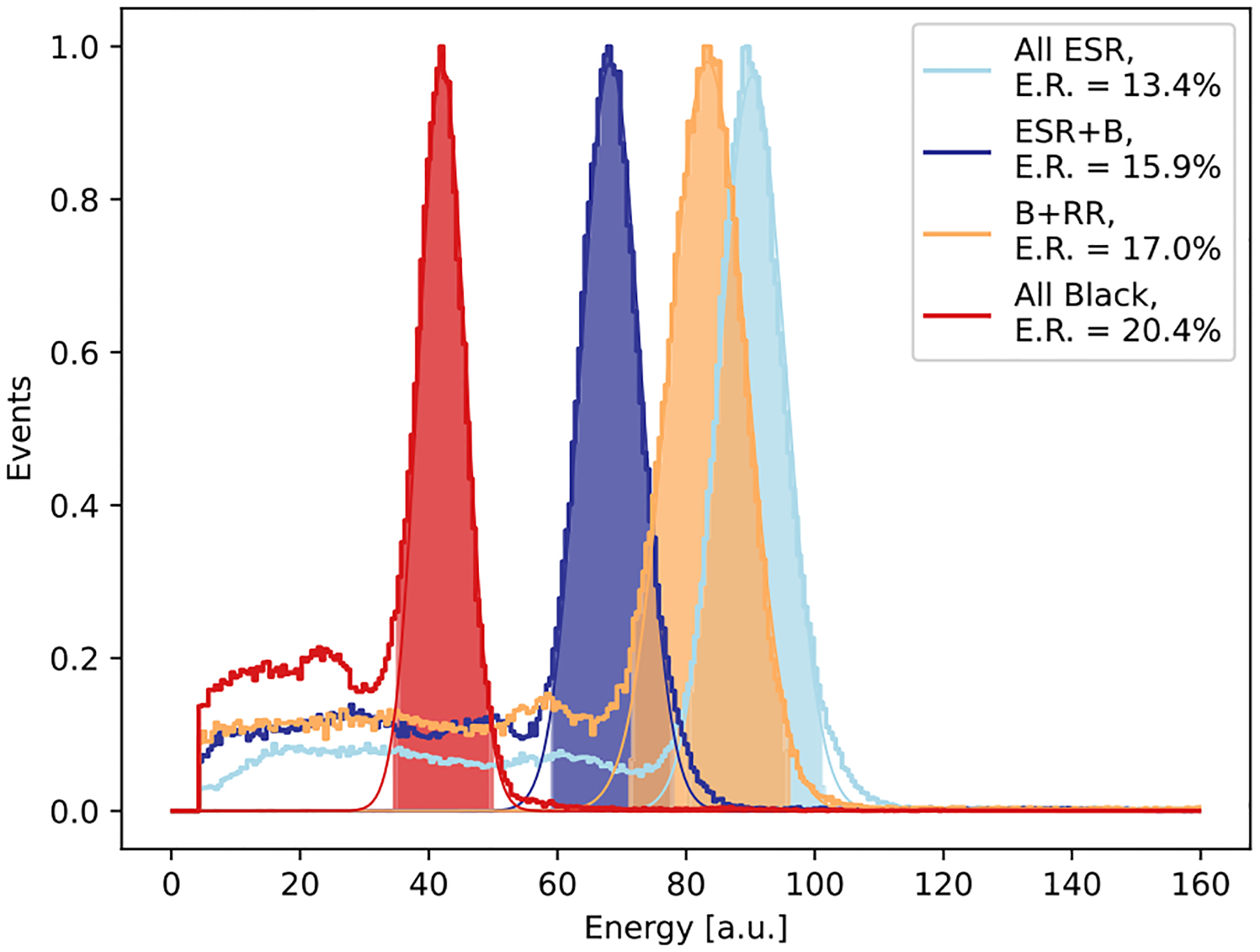
Energy spectra acquired in coincidence mode with a ^22^Na source for each semi-monolithic array, with different surface treatments applied. The spectra represent the response of the entire array, after applying gain equalization among slabs.

The energy spectra are shown in Fig. 4, together with the Gaussian fit of the photopeak and the resulting energy resolution. Note that because different bias voltage were used for the four arrays (see Table I), the photopeak positions cannot be directly compared to quantitatively assess differences in light output. However, as reasoned in Section II-C, the energy resolution values of the four arrays are comparable. As expected, the best energy resolution is achieved with the *all ESR* array (13.4%) because of the higher light output. This is followed by the *ESR*+*B* array (15.9%), while a larger degradation is obtained with *RR+B* (17.0%) and *All Black* (20.4%) arrays due to the significantly decreased light output.

**Fig. 5.**
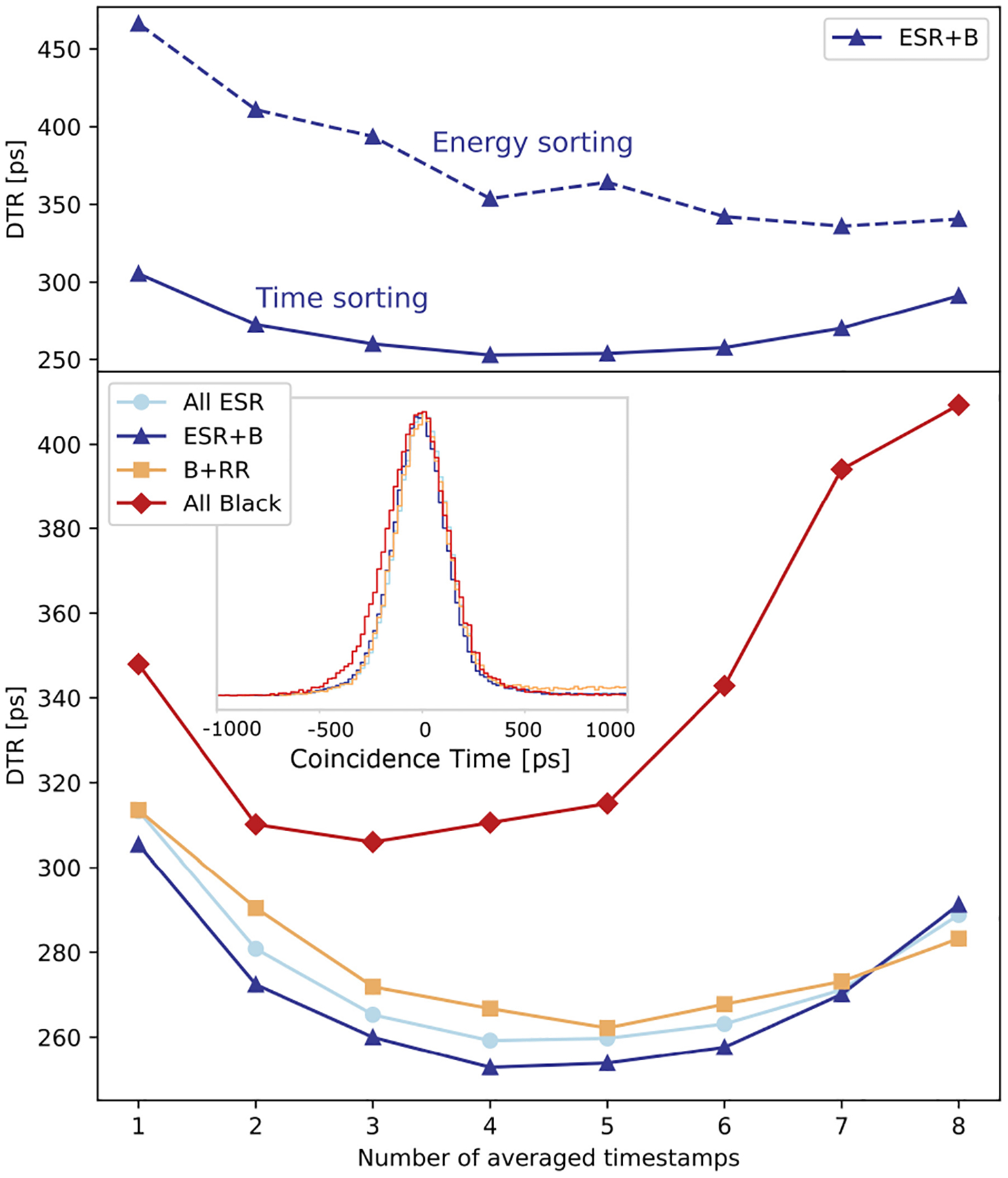
DTR as a function of the number of averaged timestamps. Top: Comparison between energy and time sorting methods using the *ESR+B* array. Bottom: Comparison between the four arrays, using the time sorting method. The inset shows the coincidence spectra obtained with the best time estimator for each array (averaging four timestamps for *all ESR* and *ESR+B*, 5 for *B+R* and 3 for *All Black*).

**Fig. 6.**
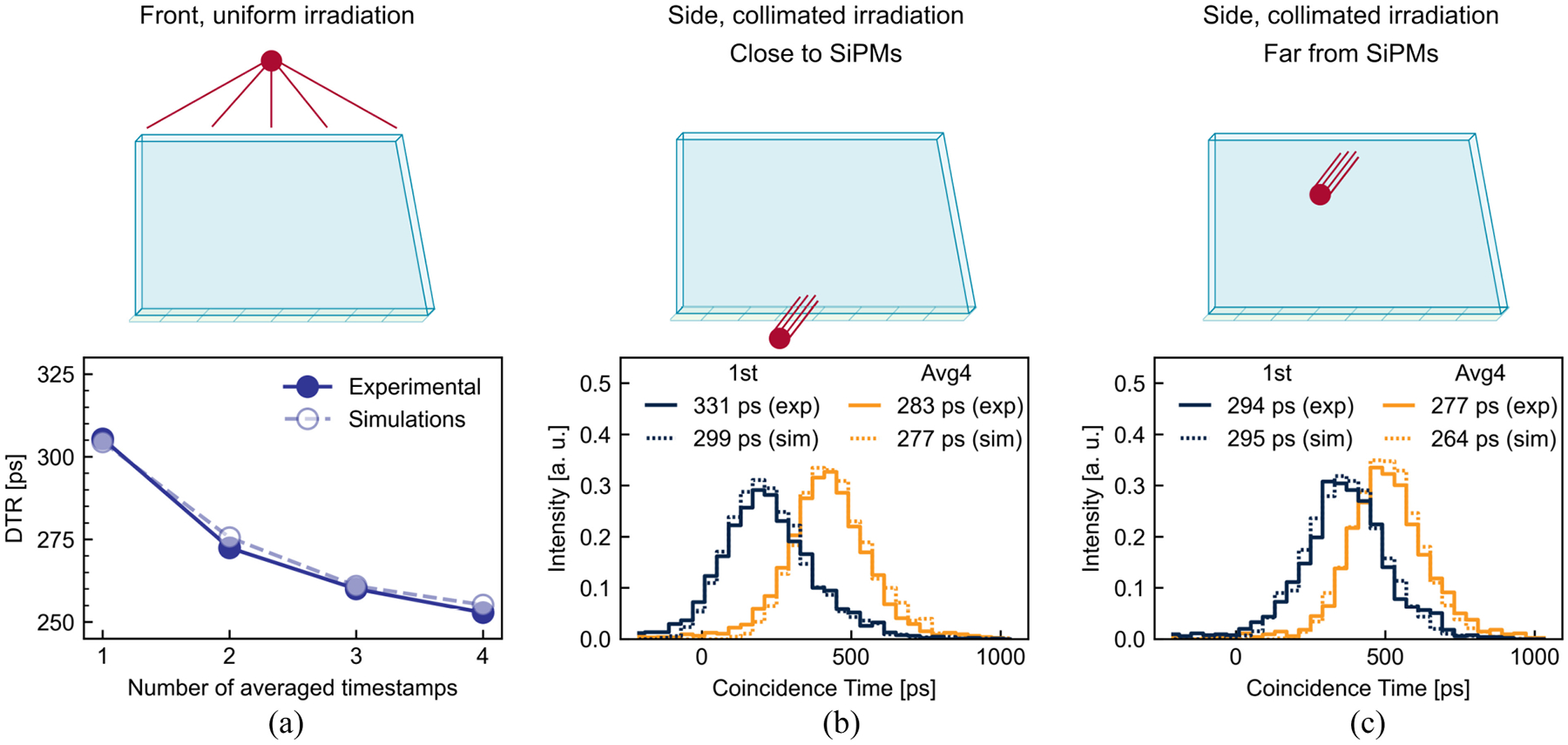
(a) Comparison of the DTR as a function of the number of averaged timestamps measured experimentally and obtained with simulations for a front irradiation. Coincidence time histograms when using the first timestamp (1st) and an average of the first four timestamps (Avg4) for a side irradiation. (b) Close to the SiPMs and (c) far from the SiPMs, with CTR values (obtained from Gaussian fits) displayed in the legends both for experimental measurements and simulations. All the results are for the *ESR*+*B* array.

Because of the light sharing among multiple SiPMs, a feature of the semi-monolithic geometry, the best timing performance is achieved when averaging multiple timestamps and weighting them accordingly to the charge collected by the corresponding SiPM. However, as noise contribution increases with decreasing charge, the correlation between time resolution and the number of averaged timestamps is nonmonotonic, and an optimal point exists. Additionally, there are two possible methods to sort SiPM hits for the averaging: by energy (with the most energetic hit giving the first timestamp) or by time (with the fastest hit giving the first timestamp). Fig. 5 (top) shows the comparison between the two sorting methods, displaying that sorting hits by time leads to better time performance. The comparison is shown only for the *ESR*+*B* array, but similar results were obtained for the other arrays. Fig. 5 (bottom) shows the comparison between the DTR as a function of the number of averaged timestamps for the four arrays, using the time sorting method. The optimal DTR is achieved when averaging the first four timestamps for the *All ESR* and *ESR*+*B* arrays, the first five for *RR*+*B* array, and the first three for the *All Black* array. Comparable timing performance was obtained for the *All ESR*, *ESR*+*B*, and *RR*+*B* arrays, while a significant deterioration is observed for the *All Black* one. Focusing on *ESR+B* being the best performing array type, Monte Carlo simulations of the DTR as a function of the number of averaged timestamps (time sorting method) is shown in Fig. 6(a) for the front irradiation, showing a trend matching the one of experimental results. The coincidence time distributions also evolve similarly in simulations and measurements as a function of the DOI and the number of averaged timestamps for a side irradiation close to the SiPMs [Fig. 6(b)] and far from the SiPMs [Fig. 6(c)]. Simulations also indicated that using the energy sorting method for the timing estimation yielded worse DTR values compared to a time sorting estimation. For instance, at the optimal number of averaged timestamps (four), the DTR in front irradiation degrades from 253 to 354 ps experimentally (Fig. 5), and simulations from 255 to 350 ps, therefore both having a degradation close to 40% and jointly highlighting that the time sorting method provides better time resolution.

A summary of the energy resolution and DTR results of the four arrays is given in Table II. Here, the reported DTR values are the ones achieved at the respective optimal number of averaged timestamps for each array type.

### B. Time Resolution as Function of Impact Position

The previous section discussed the timing performance when uniformly irradiating the crystals from the front face, summing the contributions from all events independently on the interaction position within the crystal. However, given the finite length of the scintillator crystal and the time spread of photon propagation, the interaction position of annihilation photons within the crystal can significantly affect the measured time resolution. The measurements in collimated side irradiation, scanning the array both along the monolithic and DOI direction, allowed to investigate the dependence of time resolution on the impact position. We evaluated this dependence considering three different time estimators: the first (fastest) timestamp, the first timestamp after applying time walk correction, and the optimal number of averaged timestamps previously assessed for each array type, also corrected for time walk.

**Fig. 7.**
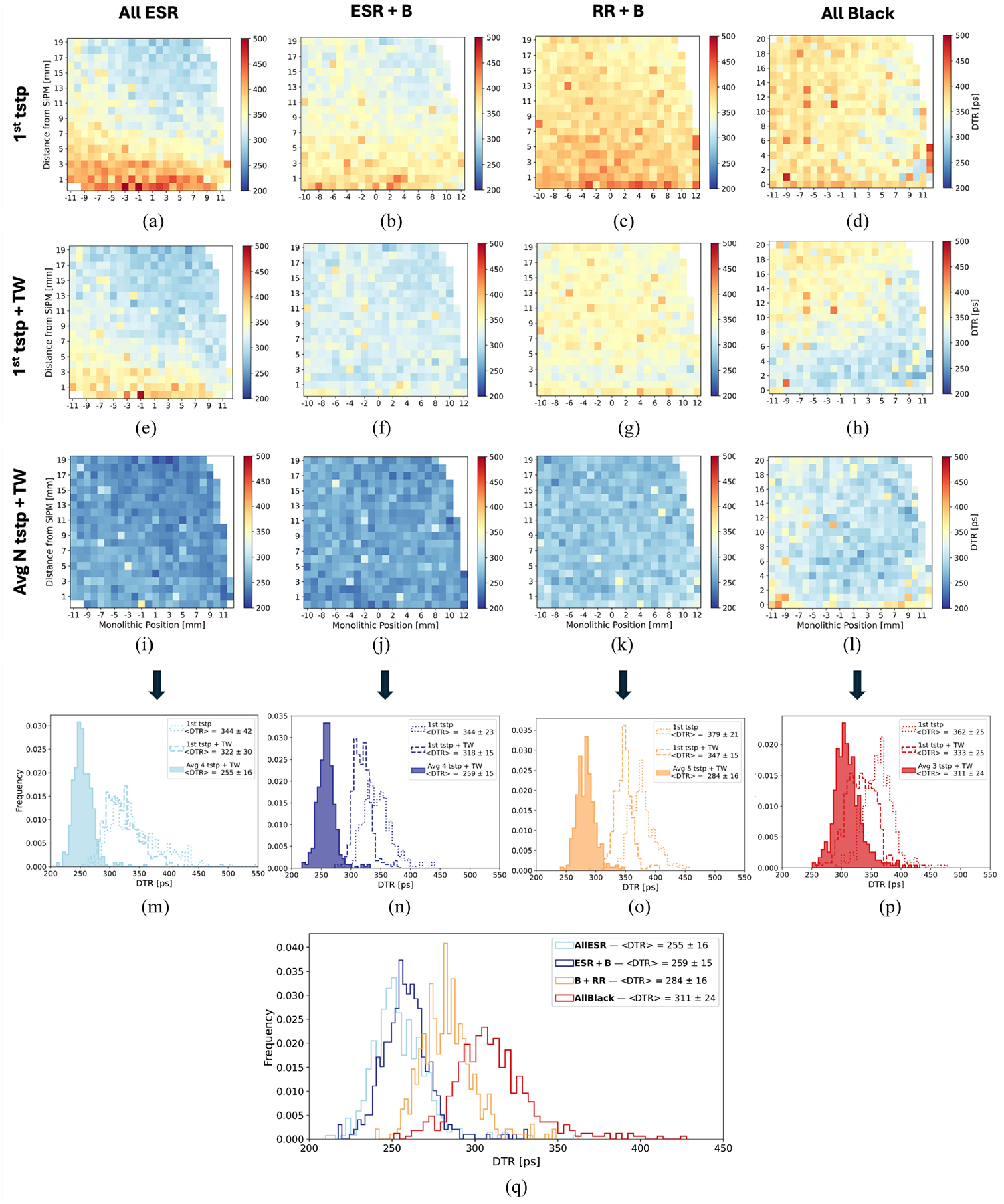
DTR dependence on the impact position. (a), (e), (i) Heatmap showing the DTR as a function of monolithic and DOI position for the *All ESR* array, using as time estimator only the first timestamp (a), the first timestamp applying the time walk correction (e), average of the first four timestamps after applying the time walk correction (i). The same it is shown for the array *ESR*+*B* (b), (f), (j), *RR+B* (c), (g), (k), and *All Black* (d), (h), (l). The 1-D distributions of the DTR values obtained with each of these three estimators are displayed for the *All ESR* (m), *ESR+B* (n), *RR*+*B* (o), *All Black* (p). In (q), the 1-D distributions of the estimator giving the best DTR for each array are compared.

Fig. 7 summarizes all the results. One can notice a strong DOI dependence when using only the first timestamp as time estimator, which is mitigated by the time walk correction and almost suppressed when performing the timestamps average. This tendency is particularly evident for the *all ESR* array, while a weaker DOI dependence was found for the *ESR+B* and *RR*+*B* arrays, and an almost uniform response for *All Black*. In Fig. 7(m)–(p), the 1-D distributions of the DTR values obtained with each estimator for the four arrays are shown together with their average and standard deviation, confirming the previous observation. For *All ESR*, the standard deviation of the measured DTR values is 42 ps when using only the first timestamp, 30 ps when applying the time walk correction, and 16 ps when averaging the first four timestamps. In the case of *ESR*+*B*, the standard deviation decreases from 23 to 15 ps with the time walk correction without further improving when averaging the timestamps. A similar behavior is also observed for *RR*+*B*, while for the *All Black* the standard deviation is constant around 25 ps in all cases.

Table III shows the comparison between the DTR measured in front uniform irradiation and the average of the DTR values at each impact position obtained in collimated side irradiation, using the same time estimator i.e., the average between the first 3–5 timestamps (according to the array type) after applying time walk. For all arrays, the results obtained in the two configurations are compatible, confirming that the time walk and timestamps averaging mitigate the DOI effect and further DOI corrections are not needed.

Interestingly, the time-DOI dependence revealed better time resolution at DOIs far from the SiPM (Figs. 6 and 7). The effect is stronger for the *All ESR* array and is almost suppressed by the timestamp averaging and the time-walk correction (Fig. 7). This trend was observed both in experimental measurements and in simulations (Fig. 6).

**Fig. 8.**
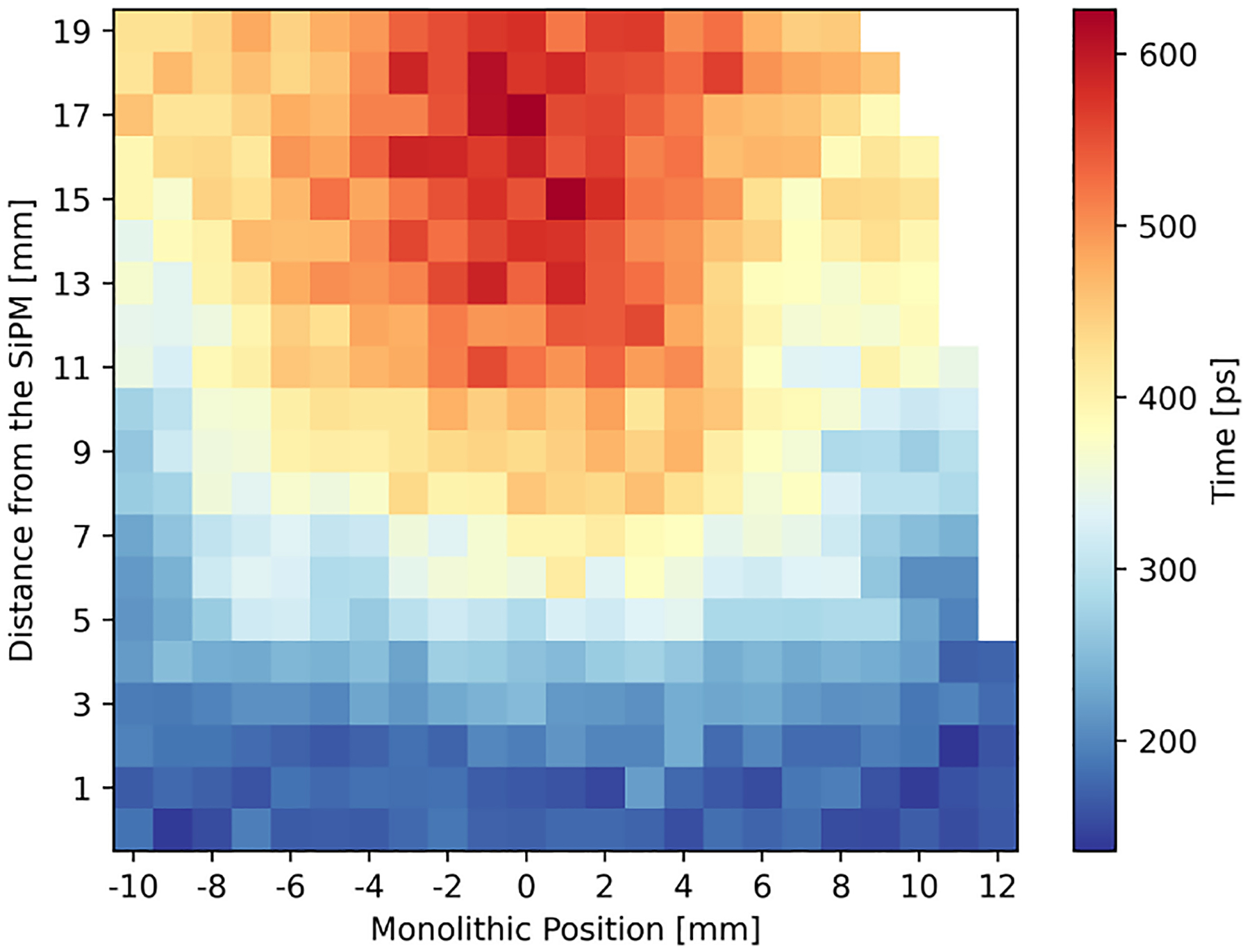
Difference between the detection time of the fastest and most energetic hits as a function of the impact position for the *ESR*+*B* array.

Because of the significant different DTR obtained when sorting the hits according to the time of arrival or the collected charge (Fig. 5, top), we analyzed the detection time difference between the fastest and most energetic hit. This distribution shows a sharp peak at 0 ps, containing about 20 %–30 % of the events—indicating that the fastest and most energetic hits coincide in these cases—and a long tail extending up to a few nanoseconds. We further investigated the correlation between the mean of this distribution and the impact position, as illustrated in Fig. 8. A strong dependence on the DOI direction was observed, with a larger time delay occurring at the DOI farthest from the SiPM. Monte Carlo simulations confirmed this trend of an increasing time delay going away from the SiPM array.

### C. Impact Position Estimation

**Fig. 9.**
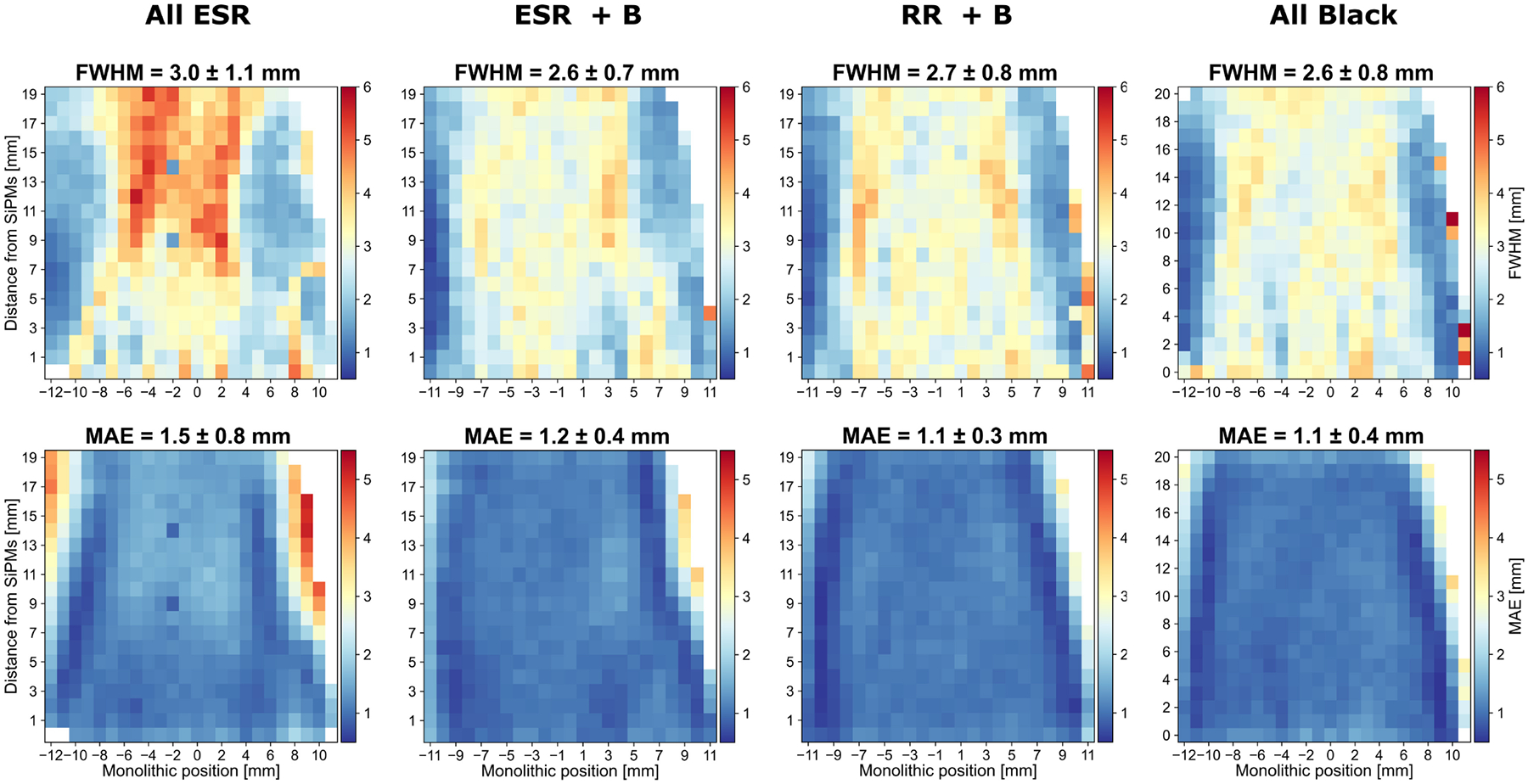
Monolithic resolution expressed in FWHM (top row) and MAE (bottom row) measured at each source position for each array. The average and standard deviation FWHM and MAE values obtained across all source positions are stated.

**Fig. 10.**
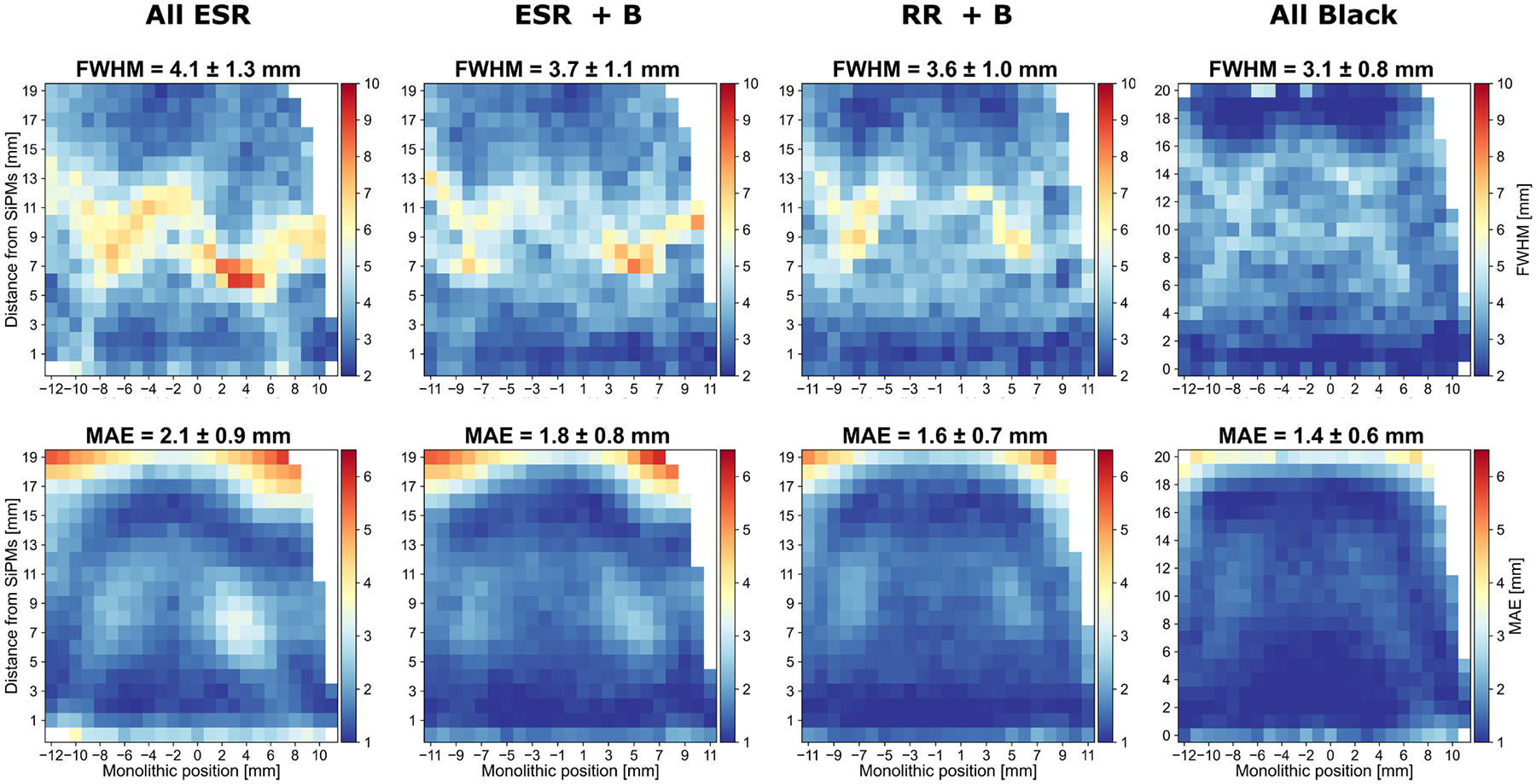
DOI resolution expressed in FWHM (top row) and MAE (bottom row) measured at each source position for each array. The average FWHM and MAE values obtained across all source positions are stated.

The compression observed in the reconstructed impact position for the *All ESR* array (Fig. 3) reflects into a worse positioning capability than arrays with at least the lateral pixelated surfaces black painted. Figs. 9 and 10 show the resolution measured along the monolithic and DOI direction, respectively, of the four arrays.

Along the monolithic direction, the average FWHM decreases from 3.0 ± 1.1 to 2.6±0.7 mm when changing from *All ESR* to *ESR*+*B*, meaning an improvement of 13 % and a reduced standard deviation of 30 %. In terms of MAE, the improvement is even more significant: 20 % better average value (from 1.5 to 1.2 mm) and a reduced standard deviation of 50 % (from 0.8 to 0.4 mm). The *RR*+*B* and *All Black* arrays behave similarly to *ESR*+*B*, without any further significant improvement.

Along the DOI direction, the FWHM for *All ESR* (4.1 ± 1.3 mm) shows about a 10% improvement with *ESR+B* (3.7 ± 1.1 mm) and *RR*+*B* (3.6 ± 1.0 mm), and a 24% improvement with the *All Black* array (3.1 ± 0.8 mm). A similar relative improvement is observed in the average MAE values. As with the monolithic direction, the enhanced average FWHM and MAE values are accompanied by a reduced standard deviation.

**Fig. 11.**
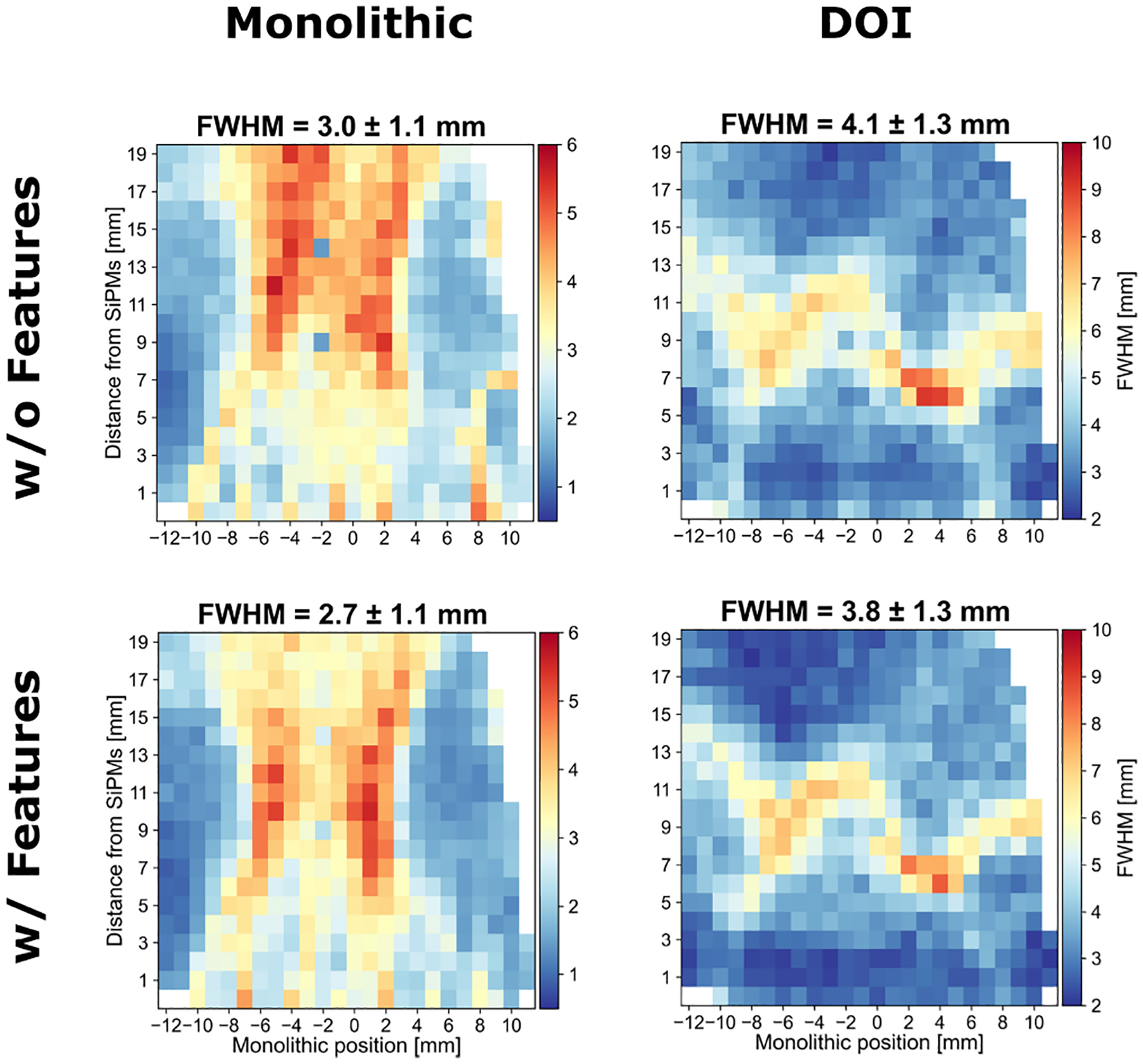
Comparison between the monolithic (left) and DOI (right) resolution in FWHM of *all ESR* array when using the standard MLP architecture, i.e., without engineered features (top row), and with engineered features (bottom row).

Previous studies demonstrated that by adding engineered features, such as the pre-estimated position and the charge variance can improve the performances of the NNs [25], [45]. Furthermore, a DOI dependence of the time difference between the fastest and most energetic hit was observed for all the arrays (see Fig. 8). After testing different combinations of the mentioned features, improved DOI and monolithic resolution were obtained for the *all ESR* array only. The comparison is shown in Fig. 11: the resolution FWHM improved by 10 % along the monolithic direction and 7 % along the DOI. Similar improvement was observed also for the MAE: 7 % and 10 % for the monolithic and DOI direction, respectively. Moreover, also the standard deviation of the MAE was reduced of about 10 % along both directions.

## IV. Discussion

The UHB scanner will be based on semi-monolithic detectors to offer good sensitivity, while providing both TOF and DOI capabilities for brain imaging, and at a moderate cost compared to other existing systems which often rely on highly-pixelated arrays. With the aim to achieve the optimum balance between TOF and positioning accuracy, we evaluated the performance of four arrays with different surface treatments: *all ESR*, *ESR*+*B*, *ESR*+*B+RR*, and *All Black*.

The *all ESR* array demonstrated the best energy resolution, as expected due to its enhanced light collection. However, ESR films while increasing light collection also introduce edge effects due to reflections on the lateral faces, significantly altering the LD. This change causes a concentration of light toward the center of the crystal, leading to a compression of the estimated impact position distribution. This effect along the monolithic direction is visible in Fig. 3, from left to right: as more black-painted surfaces are introduced, the compression decreases. *RR+B* shows smaller compression compared to *ESR+B* because the RR partially preserves the original direction of optical photons. The results on monolithic and DOI resolution are consistent with the observations above. Regarding the monolithic resolution, a significant and comparable improvement was recorded for all the arrays having at the least the lateral pixelated black painted compared to the *all ESR*: the average FWHM and MAE improved by 13% and 20%, respectively, accompanied by a reduced standard deviation across the whole array. The comparable behavior of the three arrays *ESR*+*B*, *RR+B*, and *All Black* along the monolithic direction can be explained by them having the same treatment on the pixelated lateral faces, which affect the positioning in this direction the most. However, the decreasing compression along the monolithic direction for *RR*+*B* and *All Black* suggests that also the treatment of the top surface has an impact. One possible explanation for the lack of improvement in resolution is the size of the collimator, which is 2 mm in diameter and, thus, comparable to the resolution values of 2.6 mm, limiting the achievable precision.

The heatmaps showing the monolithic (Fig. 9) and DOI (Fig. 10) reveal symmetric patterns featuring poorer resolution (FWHM) at central coordinates along the monolithic and DOI direction, respectively. Such behavior is likely related to specular reflections and light propagation effects, which are themselves dependent on the emission position. Indeed, these patterns become progressively less pronounced when ESR is replaced with either a retroreflector or black paint. Similar findings were reported in [39]. *all ESR*, *ESR+B* and *RR+B* arrays showed comparable time resolution in a range of 10 %, with *ESR*+*B* performing slightly better than the other two. Instead, by black-painting also the entrance surface of the array, the time resolution worsens by at least 20 %, similarly as for the energy resolution.

The analysis of time resolution dependence on impact position revealed better DTR at DOIs far from the SiPM. In pixelated crystals, the time resolution has also been observed to worsen when moving from the crystal end far from the SiPM to the central region, but then improves again near the SiPM, eventually surpassing the performance observed at the far end [47], [48]. This behavior has been attributed to a tradeoff between reduced photon time spread at larger distances from the SiPM and enhanced light collection efficiency near the photosensor. In semi-monolithic arrays, however, light is distributed among multiple SiPMs—even for interactions occurring near the detector surface—so the advantage of enhanced light collection may not fully compensate for the increased photon time spread, resulting in poorer time resolution close to the SiPM. The effect is more pronounced in the *all ESR* configuration, supporting the hypothesis that photon time spread due to multiple reflections plays a key role in the observed trend. However, for all surface treatments, after applying time walk correction and timestamp averaging, the DOI dependence is significantly reduced, eliminating the need for any additional DOI-based time correction.

For the above reasons, we selected *ESR+B* as the treatment for the semi-monolithic arrays we will use to build the UHB-PET scanner. Indeed, although the average monolithic and DOI resolution (FWHM) of the *all ESR* treatment approaches those of *ESR*+*B* when engineered features are included, its point-to-point resolution is less uniform, exhibiting a higher standard deviation, and notably worse at the center of the slab. These observations reinforced our decision to adopt *ESR*+*B* for the scanner. Summarizing, the *ESR+B* featured an overall DTR of 253 ± 15 ps together with an energy resolution of 15.9 ± 0.6 % when considering the whole array (before further optimization), and a position estimation accuracy of 3.7 ±1.1 mm and 2.6 ± 0.7 mm along the DOI and monolithic direction, respectively.

It should be emphasized that these will not necessarily be the final performance of the detector. While the main purpose of this work was to compare the four treatments, optimization of the selected array is still in progress. We will first validate the current performance after implementing the channel reduction readout, which, based on previous similar studies, is expected to have a minor degradation only in time resolution [30]. Positioning accuracy was already assessed using artificially summed energy signals along SiPM rows and columns to emulate this readout. For the selected *ESR*+*B* array, we plan to recalibrate the impact position estimation using a slit collimator with submillimeter width, replacing the 2 mm pinhole used in this study. This will reduce collimator-induced blurring and improve DOI and monolithic resolution measurements. This work also showed that incorporating engineered features into the MLP improves positioning accuracy by about 10% (Fig. 11). Additionally, time information was found to provide complementary insight into the interaction position. We plan to further integrate engineered features and timing with energy data to enhance accuracy. In parallel, we are exploring more advanced NN architectures, such as convolutional NNs (CNNs), which have shown improved performance in preliminary studies by better capturing the spatial structure of LDs [49].

## V. Conclusion

In this work, we evaluated the performance of four semi-monolithic LYSO-based arrays with different surface treatments. Our primary goal was to identify the type of array that offered the best tradeoff between impact position accuracy, energy, and TOF resolution, particularly for a new brain PET scanner under development as part of the UHB-PET project (a USA National Institute of Health awarded grant), which is scheduled to be assembled by the end of next year.

The chosen array features ESR on all surfaces except for the black-painted lateral pixelated ones (*ESR+B*). Compared with the *All ESR* array, which should provide the best performance in time and energy resolution, the *ESR+B* showed a small degradation only in the latter, while time resolution was preserved, if not slightly improved. A Monte Carlo simulation framework was also designed, confirming the trends of time resolution measured experimentally. An improvement on the order of 10 % in position accuracy in terms of FWHM and 20 % in terms of MAE, along both monolithic and DOI directions, was obtained with *ESR+B* compared to *All ESR* when using standard MLP architecture (i.e., without engineered features). Overall, the selected array achieved 15.9 ± 0.6 % energy resolution, 253 ± 15 ps DTR. Using NNs with MLP architecture, a position accuracy of 3.7 ± 1.1 mm and 2.6 ± 0.7 mm FWHM was measured along the DOI and monolithic directions, respectively.

This semi-monolithic detector design offers a high-performance, cost-effective solution for TOF-PET imaging of the brain, enabling enhanced precision and accessibility in neuroimaging applications.

## Figures and Tables

**TABLE I:** Optimal Combination of Bias Voltage (*V*_*b*_) and PETsys Parameters (*vth*_*t*1_*, vth*_*t*2_*, vth*_*e*_) for Each Array. The Values of *vth*_*t*1_*, vth*_*t*2_*, vth*_*e*_ Are in DAC Units

	*V*_*b*_ [V]	*vth*_*t*1_	*vth*_*t*2_	*vth*_*e*_
*All ESR*	55	5	25	20
*ESR+B*	56	5	20	8
*RR+B*	57	4	15	8
*All Black*	57	4	20	10

**TABLE II:** Summary of Energy Resolution (E.R.) and DTR Performance of the Four Array Types

	All ESR	ESR+B	RR+B	All Black
E.R. [%]	13.4 ± 1.0	15.9 ± 0.6	17.0 ± 1.2	20.4 ± 1.7
DTR [ps]	259 ± 17	253 ± 15	262 ± 12	306 ± 20

**TABLE III:** Comparison Between DTR Measured in Front Uniform Irradiation (*Front*) and in Collimated Side Irradiation (*Side*). For Side Irradiation, the DTR Values Reported Represent the Average Across the DTRs Measured at Each Position

		All ESR	ESR+B	RR+B	All Black
DTR [ps]	Front	259 ± 17	253 ± 15	262 ± 12	306 ± 20
	Side	255 ± 16	259 ± 15	284 ± 16	311 ± 24
